# molIEreVIS: exploring and interpreting the evidence behind drug repurposing predictions

**DOI:** 10.3389/fbinf.2026.1756459

**Published:** 2026-03-30

**Authors:** Amal Alnouri, Andreas Hinterreiter, Christian Steinparz, Labinot Bajraktari, Sebastian Burgstaller-Muehlbacher, Markus Bauer, Gregorio Alanis-Lobato, Marc Streit

**Affiliations:** 1 Visual Data Science Lab, Johannes Kepler University, Linz, Austria; 2 Boehringer Ingelheim RCV GmbH & Co KG, Vienna, Austria; 3 Boehringer Ingelheim Pharma GmbH & Co KG, Biberach, Germany

**Keywords:** drug repurposing, indication expansion, interpretability, knowledge graph, visualization

## Abstract

**Introduction:**

Finding new uses for existing drugs, known as drug repurposing, is a widely adopted drug development strategy in the pharmaceutical industry. Computational drug repurposing leverages vast biomedical data to prioritize repurposing candidates. Once these candidates are prioritized, domain experts face the burden of evaluating their true potential.

**Methods:**

In this work, we propose a visualization-based approach to address this challenge for a multimodal class of computational drug repurposing, where heterogeneous evidence modalities are integrated. We conducted a design study in close collaboration with domain experts, from which we derived a domain abstraction of the expert assessment process. Grounded in this abstraction, we developed an interactive visualization approach that explicitly models the expert reasoning process. We applied the proposed approach to create a prototype implementation, molIEreVIS, in the context of an operational drug repurposing pipeline. We used this prototype to collect qualitative feedback from domain experts actively engaged in assessing computational drug repurposing candidates.

**Results:**

The results demonstrate the potential of our approach to support insights and reasoning in this process and reveal directions for enhancements and future work.

## 1 Introduction

*De novo* drug development is known for its lengthy timelines, high attrition rates, and escalating costs (Hill and Richards, 2021; Ashburn and Thor, 2004; Pushpakom et al., 2019; Pinzi et al., 2024). These challenges have led drug developers to find new uses for existing drugs, a process known as drug repurposing (DR), and offers the potential for a less risky development process, shorter timelines, and significantly lower costs (Ashburn and Thor, 2004; Pushpakom et al., 2019; Pinzi et al., 2024). Drug repurposing used to be opportunistic, which is the accidental discovery of an existing drug’s activity in a new therapeutic context (Pushpakom et al., 2019; Hamid et al., 2024).

In the era of big data, the vast availability of data from diverse sources has marked a turning point in biomedical research (Cremin et al., 2022). Coupled with advances in computational approaches, nowadays, computational drug repurposing formulates repurposing hypotheses by retrieving, integrating, and analyzing such data sources to uncover the complex indirect relationships between drugs, biological targets, and diseases (Pinzi et al., 2024; Saranraj and Kiran, 2025). It has transformed the traditional opportunistic one-hypothesis-at-a-time process into a systematic and comprehensive exploration of possible repurposing opportunities (Liu et al., 2013).

Still, these opportunities are less reliable than the ones prioritized by traditional approaches Cavalla (2019), requiring careful assessment by domain experts to determine their true potential. This assessment involves a detailed investigation of the biomedical evidence considered in the prioritization process. In this work, we address this challenge through a visualization-based approach grounded in a design study we carried out in close collaboration with domain experts.

To narrow down the scope of our contribution, it is necessary to consider the wide variety of computational drug repurposing approaches, that differ substantially based on their underlying data sources (Tanoli et al., 2025; Cousins et al., 2024; Kulkarni et al., 2023; Jarada et al., 2020; Pushpakom et al., 2019). Table 1 summarizes major repurposing approaches and their associated data modalities. Previous reviews (Pinzi et al., 2024; March-Vila et al., 2017) have also emphasized the need to integrate different data modalities toward a more comprehensive modeling of the complex interaction between biomedical entities, and to overcome the inherent weaknesses of each when used alone. This work addresses the assessment challenge for the multimodal class of computational drug repurposing, investigating how diverse lines of evidence can be jointly explored and evaluated.

**TABLE 1:** Overview of computational drug repurposing approaches.

Based on	Data source	Description	Representative references
Structures	3D structures of biological targets	Molecular docking and binding affinity estimation	Ngernsombat et al. (2024); Khan et al. (2025); Lv et al. (2024); Iqbal et al. (2025)
Signatures	Molecular signatures derived from omics data (e.g., transcriptomics, proteomics)	Expression signature matching	Sun et al. (2025); Nikolakis et al. (2025)
Genetic associations	Genetic association data (e.g., Genome-wide association studies (GWASs))	Identification of disease-associated genetic variants	Seagle et al. (2025); Yu et al. (2024)
Networks	Interaction networks and multimodal knowledge graphs	Network propagation and link prediction	Chandak et al. (2023); Zhang et al. (2022)
Literature	Scientific literature	Literature mining for relationship discovery	Henry and McInnes (2017); Preiss (2025); Sikirzhytskaya et al. (2025)
Health data	Real-world health data	Real-world evidence mining	Adamek et al. (2024); Zang et al. (2023)

Previous research has recognized the importance of assessing computational drug repurposing candidates. In general, studies that deliver computational repurposing candidates include a subsequent validation step in which false positives are excluded and the overall performance of the repurposing method is evaluated. Table 2 summarizes the validation strategies reviewed by Brown and Patel (2018) and Pillai and Wu (2024). Several studies have been dedicated to developing automated validation approaches (Ozery-Flato et al., 2020; Santamaría et al., 2021; Schatz et al., 2021; Nunes and Pesquita, 2024). Similar to the aforementioned strategies, these approaches use existing biomedical data to contextualize the repurposing candidates with additional evidence from existing knowledge. For instance, Nunes and Pesquita (2024) validate a single drug repurposing candidate by finding k -shortest informative paths between the drug and the disease in an independent knowledge graph. A key feature of this validation literature is that it focuses solely on the outcomes of the validated methods rather than providing insights into the process behind them, and relies on external data sources, rather than leveraging the evidence identified by the methods themselves. Furthermore, it often summarizes findings into quantitative metrics, which, while useful, lack explanatory depth and still requires expert interpretation.

**TABLE 2:** Overview of computational drug repurposing validation strategies.

Strategy	Automated	Description
Retrospective clinical analysis	Yes	Searching real-world health data to identify off-label usage or clinical trial evidence
Literature mining	Yes	Analyzing biomedical literature to verify drug–indication connections
Benchmark datasets analysis	Yes	Validating candidates against benchmark datasets
Public database search	No	Manually searching public database (e.g., DrugBank (Wishart et al., 2008)) for drug–indication connections
Literature search	No	Manually searching through relevant scientific literature

In contrast, other works focus on developing self-explainable computational drug repurposing methods. For instance, Gurbuz et al. (2022) and (Jiménez et al., 2024) developed inherently transparent repurposing methods based on knowledge graphs. In their methods, candidates are directly underpinned by explicit paths in the knowledge graph, which makes them interpretable by domain experts. Closely related research to this work approaches this problem from a visualization and human–computer interaction perspective, where a visualization design is proposed to support domain experts in assessing the prioritized candidates. In this context, we report two works that address the problem for the network-based repurposing approach, where the candidates are prioritized by a graph neural network (GNN). Wang et al. (Wang et al., 2022; Huang et al., 2024; Wang et al., 2021) developed DrugExplorer, which provides path-based explanations using GraphMask, presenting them as both individual paths and aggregated paths (meta-paths). They also proposed a novel visualization design, MetaMatrix, with interactive features that help domain experts organize and compare explanation paths at different levels of granularity to generate domain-meaningful insights. Similarly, HypoChainer (Jiang et al., 2025) traces paths in the knowledge graph to explain GNN predictions. HypoChainer incorporates LLMs as a natural language interface for graph exploration. Experts can ask a retrieval-augmented LLM about the rationale behind a prediction, and the response is based on existing KG connections. In an iterative process, experts and the LLM collaboratively construct structured reasoning paths. Finally, the workflow filters predictions based on alignment with KG-supported evidence.

While previous works proposed visualization designs to support domain experts in network-based drug repurposing, our design study addresses this challenge for multimodal drug repurposing. We summarize our contributions as follows:

- A domain abstraction of expert assessment in multimodal computational drug repurposing.
- A visualization-based approach that explicitly models how domain experts explore, interpret, and validate drug repurposing candidates.
- A novel evidence provenance visualization that enables experts to trace evidence states through successive transformations.
- A qualitative user study, demonstrating the potential of the proposed approach to support expert insight generation and reasoning.

## 2 Materials and methods

This section provides a detailed overview of the methodologies we followed and materials we utilized to derive and evaluate the proposed approach.

### 2.1 Design study outline

A team with complementary backgrounds in visualization and bioinformatics carried out the design study, including computational drug repurposing experts who acted as domain intermediaries. The study followed a two-stage process:

1. The first stage began with a workshop that laid the groundwork for a rough domain characterization, introducing the workflow of multimodal drug repurposing pipelines, target users, challenges, and practices in assessing computational drug repurposing candidates. From this point onward, the stage adopted an iterative approach. Insights gained from the workshop informed initial domain and task abstractions, as well as early conceptual design sketches, all of which were refined based on expert feedback during regular meetings.
2. In the second stage, we instantiated the resulting design in a prototype implementation grounded in an operational drug repurposing pipeline. The prototype was then tested by representative target users to validate and reflect on the proposed design.

### 2.2 Domain abstraction

This section presents our abstraction of the study domain, including the target users, their practices, and data.

Through the close collaboration with domain experts, and informed by prior literature (McDonagh et al., 2024; Steyaert et al., 2023; Ivanisevic and Sewduth, 2023; López de Maturana et al., 2019; Thorman et al., 2024; Li and Ke, 2025), we model a multimodal drug repurposing pipeline, independent of how it is implemented, using the following four conceptual evidence transformation boundaries:

1. Evidence collection: *Qualitative evidence* is collected from diverse heterogeneous data sources.
2. Evidence quantification: Qualitative evidence is transformed into *quantitative evidence*, through manual feature extraction, learned modeling, or a combination of both.
3. Evidence harmonization: Quantitative evidence is aligned into a common representational space producing *harmonized evidence*, such that disparate modalities become comparable. This can be achieved, for instance, through saturation, normalization, or a shared latent space.
4. Evidence integration: multiple evidence modalities are integrated into a *consensus score* through methods spanning simple rule-based ones to advanced model-based ones. Regardless of the underlying method, evidence integration—either implicitly or explicitly—yields the *contribution* of each evidence modality to the consensus score.

These boundaries give rise to *data artifacts* that form a basis for a supportive visualization design. Table 3 summarizes these transformation boundaries and their corresponding data artifacts. The obtained consensus score is used to rank the repurposing hypotheses.

**TABLE 3:** Evidence transformation boundaries and data artifacts.

Boundary	Methods	Data artifacts
Evidence collection	—	Qualitative evidence
Evidence quantification	Rule-based, model-based	Quantitative evidence
Evidence harmonization	E.g., saturation, normalization, projection in shared latent space	Harmonized evidence
Evidence integration	Rule-based, model-based	Consensus score, per-stream contribution

Some pipelines realize all boundaries explicitly, particularly those that quantify each evidence modality independently, then perform a distinct harmonization step before final integration. Other pipelines collapse the harmonization boundary directly into a model-based integration process, or they collapse both quantification and harmonization boundaries by applying early integration directly to qualitative evidence. When a pipeline collapses a boundary, the corresponding data artifacts also collapse.

Notably, our observations across prior literature suggest that this model is not specific to drug repurposing, but rather constitutes a general model for multimodal hypothesis generation across diverse computational drug discovery domains, including target prioritization and biomarker discovery.

The target users in our study are drug repurposing experts, ranging from wet-lab biologists with no familiarity with the computational pipeline processes to computational biologists with a conceptual understanding of its processes. The computer-aided repurposing process begins by selecting a single repurposing context (e.g., a drug) and querying the pipeline for candidates within that context. The post-prioritization assessment aims to shortlist candidates that deserve progression to subsequent laboratory validation. During this process, the former user group is able to examine prioritized hypotheses to identify promising candidates, while the latter user group is able to perform more in-depth computational analyses and communicate their results to the former group to support collaborative decision-making. The domain experts’ reasoning model for the assessment process can be summarized in the following three-stage analysis process, where an expert engages at a stage based on their expertise:

1. Exploration: Experts screen the ranked list of hypotheses in conjunction with the consensus score, and would further benefit from examining per-stream contributions. At this stage, they look for potential inconsistencies, such as misalignment with domain expectations, negligible differences in consensus score magnitudes, or conflicting signals across evidence streams. Beyond the ranking, experts engage in higher-level reasoning by interpreting consensus scores within domain ontologies (e.g., disease ontologies). Furthermore, discussions with experts revealed that, when applicable, those who are familiar with the pipeline processes would benefit from examining alternative scenarios at this stage, for example, by disabling specific data streams or adjusting their relative influence. Such reasoning allows experts to mitigate biases introduced by uneven evidence, including cases where literature-based evidence is sparse, as in the study of “first-in-class” drugs with limited prior research. In addition, it supports robustness validation. The outcome of this stage is a shortlist of candidate hypotheses for further investigation.
2. Interpretation: Experts examine the qualitative evidence of the shortlisted candidates to assess whether it provides a coherent and sound rationale for the candidate. Discussions with experts revealed that those familiar with the pipeline processes would benefit from the ability to follow evidence through the explicit transformation boundaries realized by the pipeline, as this enables more informed and transparent judgments.
3. Validation: In line with established validation practices in the literature, experts validate the shortlisted candidates by consulting external knowledge, typically a biomedical network. This stage contextualizes the evidence in a broader domain understanding.

### 2.3 Design goals

Guided by the outlined domain abstraction, we conclude the following design goals to embody the experts’ reasoning model and needs.

**DG1** *Support coarse-to-fine analysis* The design should mirror the coarse-to-fine staged process followed by domain experts to match their mental model and create an intuitive experience. Furthermore, such a design in addition to adopting detail-on-demand principle in the individual stages, aligns the interface with the user’s immediate tasks, reduceing cognitive load and increaseing accessibility to experts who are less familiar with the pipeline processes and may prefer to engage only at a coarse level of analysis.

**DG2** *Directed analysis from query to candidate* The analysis should start from the analysts’ query and allow them to constrain views to their shortlisted candidates.

**DG3** *Support evidence investigation* Users should be able to examine the individual pieces of qualitative evidence. In addition, the design should allow tracing evidence provenance from per-stream contributions in the consensus score back to the underlying qualitative evidence, through the explicit transformation boundaries realized by the pipeline.

**DG4** *Support consistency validation* Validating the consistency of the biological relevance between the query and the candidate in the context of broader curated knowledge should be an integral part of the design.

**DG5** *Support “what-if” analysis* The design should allow experts to explore how rankings change under alternative “what-if” scenarios.

**DG6** *Support ontology-informed analysis* The design should support the interpretation of results within domain ontologies.

### 2.4 Operational context: molIEre

Drug repurposing can be approached from three angles (Parisi et al., 2020; Thakur et al., 2024): (*i*) a **disease-centric** approach finds untreated diseases that share underlying biological pathways with an established drug indication (the medical condition the drug is indicated for); (*ii*) a **drug-centric** approach broadens the usage of an established drug by connecting it to a new biological target and its associated indication; and (*iii*) a **target-centric**, approach—also known as **indication expansion (IE)** (Tanoli et al., 2025)—pairs an established drug and its known biological target with a newly identified indication, where the drug’s mode of action (MoA) also has a beneficial effect.

We evaluate our approach in the context of an operational indication expansion pipeline, called molIEre, which is an unpublished pipeline in active use at *Boehringer Ingelheim* ^1^. The pipeline is initiated using molecular targets of a known mode of action (MoA) and disease terms mapped across multiple ontologies. It integrates multiple data streams, each of which consumes a different data source to collect supporting or opposing evidence for the presumed association between the input MoA and the input disease. The demonstrated version of the pipeline in this paper consumes data from three omics sources (genomics, transcriptomics, proteomics) and one non-omics source (scientific literature).

**FIGURE 1:**
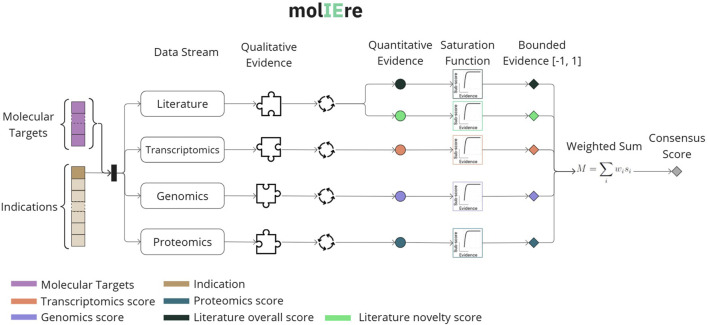
The operational indication expansion pipeline that contextualizes our study instantiation. The pipeline binds a consensus score $\left(M_{\text{score}}\right)$ to a potential connection between a known MoA and a new indication. Using the molecular targets of an MoA and a disease term, it collects qualitative evidence from four independent data sources, which is subsequently quantified. Literature evidence yields two quantitative values: *novelty*, derived from recent publications, and *overall*, derived from all publications. The quantitative evidence is then saturated to produce a scalar harmonized evidence in the range [−1,1] . Finally, the pipeline aggregates the bounded evidence through a weighted sum, yielding the final $M_{\text{score}}$ .

molIEre quantifies each data source independently, then bounds the quantitative evidence into a shared scale. Afterwards it integrates evidence modalities into a consensus score $\left(M_{\text{score}}\right)$ using an expert-designed weighted sum model. Figure 1 illustrates the complete workflow of molIEre which explicitly realizes all the transformation boundaries in Table 3.

### 2.5 Technical approach

The design instantiation, molIEreVIS, is a full-stack application composed of a frontend and a backend. molIEreVIS requires as input the data artifacts produced by molIEre, for which we designed a PostgreSQL database. The backend is a FastAPI server, that can query the PostgreSQL database. Additionally, to support the validation stage logic (see Section 3.1.3), the backend dynamically queries PrimeKG (Chandak et al., 2023), a knowledge graph stored in a Neo4j database, to retrieve paths between entities and group them under meta-paths. The frontend of molIEreVIS was developed with React, and we used D3.js to create custom visualizations. The source code is available at https://github.com/jku-vds-lab/molierevis, and our live demo can be tried out at http://molierevis.jku-vds-lab.at/.

One implementation challenge we encountered originated from the fact that molIEre candidate indication names are drawn from the MeSH (U.S. National Library of Medicine, 2025) ontology, whereas users need to query PrimeKG using these candidate names for validation. This required mapping disease names between MesH and the MONDO disease ontology (Shefchek et al., 2019) used by PrimeKG. To address this, we implemented a fuzzy matching approach based on string similarity between disease names. For a selected MeSH disease, we compute similarity scores against MONDO disease names in PrimeKG and retain candidates above a predefined threshold. For example, the MeSH disease “*Pneumonia, Bacterial*” is matched to the MONDO disease “*bacterial pneumonia*”, despite differences in word order and punctuation. These candidate matches are presented to the user, who can refine the selection by deselecting unrelated suggestions or selecting the most appropriate ones as needed.

### 2.6 User study setup

We conducted a qualitative user study with three domain experts occupying different functional roles at *Boehringer Ingelheim*. Their experience in the field ranges from two to 4 years, and all have experience in reading and creating charts. The participants’ familiarity with molIEre processes spans from good conceptual understanding to none, with **P1**, **P2**, and **P3** ordered accordingly. None of the study participants were involved in the design of our solution.

Before conducting the main user study, we ran a pilot study with one participant who was not involved later in the main study. The participant is a senior bioinformatician with 3 years of experience in the field, and has no connection to *Boehringer Ingelheim*. The pilot study helped us evaluate the preliminary version of our study protocol, and led us to refine both the study setup, as well as molIEreVIS design, particularly the design for adjusting the weights during the exploration stage (see Section 3.1.1).

We designed the study to evaluate the usefulness of molIEreVIS defined by Nielsen Norman Group (Nielsen, 2012) as a combination of: *usability* (how easily and effectively users can interact with the system) and *utility* (whether the system fulfills a specific need or solves a problem).

The study was conducted in isolated 70-min sessions, one for each participant. To assess usability, we took structured notes throughout the sessions that documented usability difficulties, such as hesitation, backtracking, minor or repetitive need for guidance, misunderstandings, or elements overlooked. Each observation was linked to a specific component where it occurred. To assess utility, we observed how the participant’s reasoning relied on molIEreVIS during an open-ended exploration. We noted behaviors such as referring to visual cues, integrating information across multiple charts or stages, making comparisons, and attempting to identify patterns. Utility was also assessed through open-ended questions (available in the Supplementary Material) in which participants reflected on their experience and whether the system would be a helpful addition to their existing workflow. The session had the following structure:

1. Briefing and personal information: The participant was first briefed on the study’s purpose and structure, and provided basic personal information.
2. Onboarding and atomic tasks: We decomposed each stage into independent sections. For each section, the participant first underwent a targeted onboarding. Then, the participant was asked to perform an atomic task focused on that specific section. These atomic tasks were designed to have a specific goal, so that it was clearly defined when a task was finished.
3. Open-ended exploration: The participant freely engaged with molIEreVIS in an open-ended exploration.
4. Feedback questions: The participant answered open-ended questions, offering feedback about molIEreVIS utility.

**FIGURE 2:**
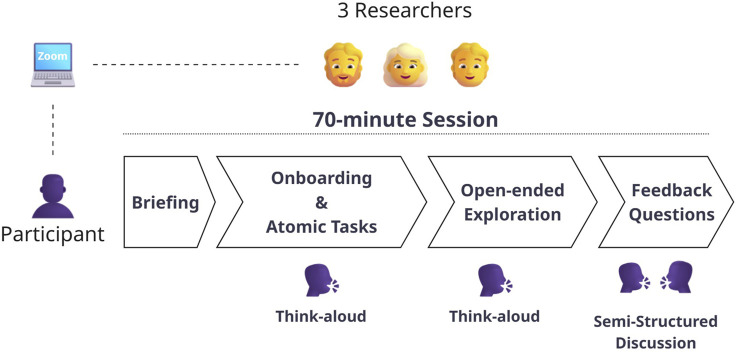
For the user study, participants took part in individual sessions, each lasting approximately 70 min and monitored by three researchers. Sessions were conducted via Zoom and structured into four stages. Both *Onboarding and Atomic Tasks* and *Open-ended Explorations* followed a think-aloud protocol. In contrast, *Feedback Questions* were conducted as a semi-structured discussion, guided by a set of predefined questions.

The study was conducted in online sessions via Zoom. For the onboarding parts, the researcher shared their screen with the participant, while during task completion and exploration, the participant shared their screen. We employed a think-aloud protocol during all sessions. Guidance was provided only when necessary or upon request. Figure 2 summarizes a session structure.

## 3 Results

In this section, we present our results, including the design instantiation and the user study.

### 3.1 Design instantiation

In this section, we present molIEreVIS, our design instantiation. The implementation of molIEreVIS is tailored to molIEre, the operational pipeline for which it was developed. However, the visualization concepts adopted for the molIEre use case are adaptable to other pipelines, particularly those that explicitly realize all evidence transformation boundaries and quantify the collective evidence per stream in a scalar value. We discuss this adaptability in more detail in Section 4.4.

**FIGURE 3:**
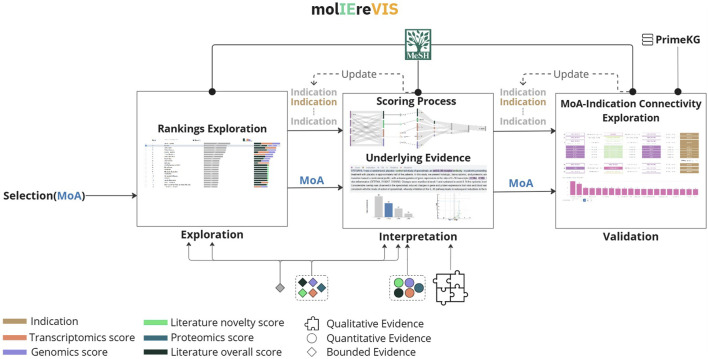
An overview of the designed multistage visualization workflow. The workflow starts by anchoring the analysis to a selected MoA. The user then narrows the focus to a specific indication, which can be updated at any time using the integrated MeSH disease ontology. The analysis proceeds through three stages: *Exploration*—molIEre’s results can be explored, visualizing both $M_{\text{score}}$ and the bounded evidence produced by saturating the quantitative evidence; *Interpretation*—molIEre’s scoring process and its underlying evidence are revealed, visualizing both the quantitative and the qualitative evidence; *Validation*—an MoA–Indication connection of interest is validated by exploring established paths between the MoA and the corresponding disease in an independent knowledge graph.

**FIGURE 4:**
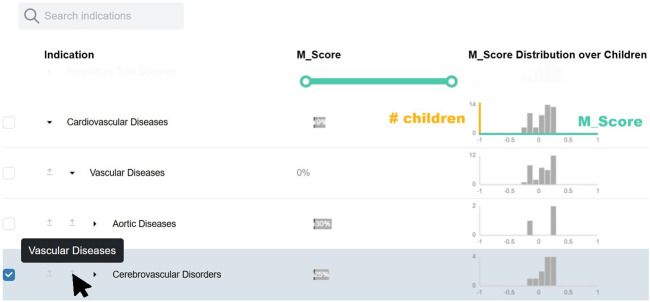
Users can select candidate indications of interest through the MeSH disease ontology, displayed as an expandable tree view. Each row represents a node in the ontology and can be expanded to reveal its child nodes. Every node is annotated with its corresponding $M_{\text{score}}$ and a sparkline showing the distribution of $M_{\text{score}}$ across its descendant nodes down to the leaves. On-demand hints associated with indentation levels help users easily identify parent–child relationships, even when nodes are expanded far from their parents.

In alignment with **DG1**, we designed molIEreVIS as a three-stage workflow: exploration, interpretation, and validation (Figure 3). At the beginning of the analysis, the user is required to select an MoA of interest from a searchable list (**DG2**). In addition, the analysis is constrained to a specific indication of interest (**DG2**), which can be selected from either the ranking table or from a separate menu exposing the Medical Subject Headings (MeSH) disease ontology (U.S. National Library of Medicine, 2025) (**DG6**), which is widely used for literature indexing. We visualize the disease ontology as an interactive, expandable tree view (Figure 4), where users can explore level by level, with the ability to search. Each node in the tree is annotated with the consensus score from molIEre $\left(M_{\text{score}}\right)$ . For each node with children, the $M_{\text{score}}$ distribution among descendant nodes spanning all levels down to the leaves is displayed in a dedicated column of sparklines. The resulting histograms highlight promising branches where high-ranked indications can be found.

In the following subsections, we outline our design for the different stages in molIEreVIS, including the basic interaction with the visual elements.

#### 3.1.1 Exploration stage

For the ranking list visualization, we adapt the visualization technique proposed in LineUp (Gratzl et al., 2013), designed to represent rankings based on heterogeneous attributes. LineUp adopts a column-based view in which each column corresponds to an attribute, with its values visualized as bar charts. Multiple attributes can be combined using a weighted sum by dragging and dropping their columns, and the resulting score is shown as a stacked bar chart.

A key distinction in our data compared to typical LineUp use cases is the presence of negative attribute values, which represent opposing evidence. To handle this, our bar charts use a zero pivot, with negative values extending to the left.

We limited the visualization and interactivity offered in LineUp to keep our visualization focused and task-oriented. We describe our design for the ranking table in the following points:

-
$M_{\text{score}}$ *column*: A bar chart column showing $M_{\text{score}}$ .
- *Detailed score column*: A stacked bar chart column to break down $M_{\text{score}}$ by data stream contribution.
- *Data stream toggling*: An option for the user to selectively toggle data streams on or off, thereby adjusting those that govern the rankings (**DG5**).

**FIGURE 5:**
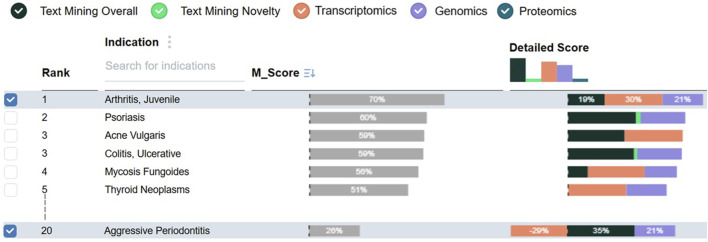
Indication ranking table displaying $M_{\text{score}}$ column and *Detailed score* column breaking down the consensus score into contributions from individual data streams. The 20th indication shows the visual encoding for the negative contribution from the transcriptomics data stream.

Figure 5 shows the ranking table for *anti-IL1RL2*, which is a known MoA for the treatment of *Psoriasis*. Consistent with domain knowledge, *Psoriasis* appears among the top candidate indications.

**FIGURE 6:**
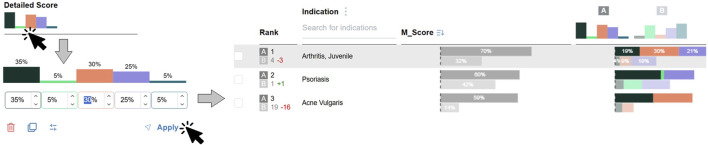
Clicking the weight bar chart in the *Detailed score* header opens a pop-up dropdown that allows users to adjust existing weights, add new ones, and select a weight set as a reference for ranking comparison. Each newly defined weight set generates an additional $M_{\text{score}}$ bar chart alongside the default ones in the ranking table, enabling direct observations for weight influence.

The stage also allows users to adjust data stream weights and observe their impact on the rankings (**DG5**). As shown in Figure 6, users can add a new set of weights by interacting with the weights bar chart in the *Detailed score* header. A pop-up drop-down appears, allowing weight values to be entered directly into text boxes. Each weight set is visualized as a shaded bar chart in the header and is labeled with a letter for easy reference. Up to five sets of weights can be added.

When a new weight set is added, a new $M_{\text{score}}$ is calculated for each indication. These are displayed as shaded bars and stacked bars in the ranking table. Each additional bar is aligned with the corresponding weight set letter label in the *rank* column, which also visually encodes ranking changes as numerical green annotation for increases, and red for decreases.

#### 3.1.2 Interpretation stage

**FIGURE 7:**
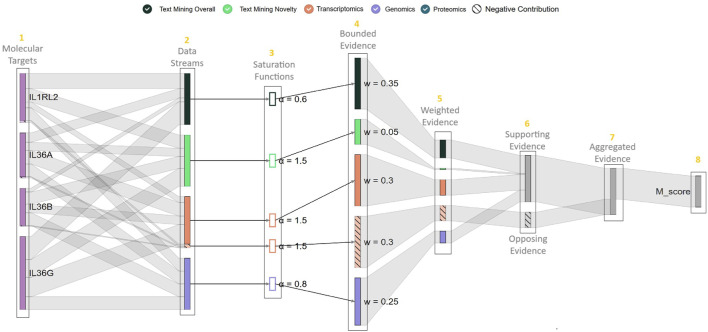
The evidence flow diagram designed for tracing the evidence during the $M_{\text{score}}$ calculation process. The diagram consists of layers with nodes and links between nodes that trace the flow and transformation of evidence across layers. It also exposes the weight and saturation parameters queried by the molIEreVIS backend from the PostgreSQL database. The figure shows the diagram for the association between *anti-IL1RL2* and *Psoriasis*.

In line with **DG1** and **DG3**, we designed the **evidence-flow diagram** shown in Figure 7. It consists of several layers arranged from left to right, comprised of nodes with links between them tracing evidence across its transformation boundaries realized by molIEre (see Figure 1).

Each node in layer 1 belongs to a molecular target of the MoA of interest, and each node in layer 2 represents the collective qualitative evidence of a data stream. The extent of hatching on a node reflects the presence of opposing evidence. Up to layer 2, the evidence collected from different data streams is not yet harmonized and therefore not comparable. For this reason, we treat the data stream nodes in layer 2 independently, giving each the same fixed size. Meanwhile, the links between the two layers represent how the total normalized evidence collected by each data stream is distributed among the targets. Consequently, the target node size represents the aggregated normalized support it receives across data streams.

Layer 3 shows the different saturation functions applied to the evidence based on its source. After saturation, layer 4 corresponds to the harmonized evidence, where the size of the node represents its bounded value. Subsequently, evidence integration starts at layer 5, the harmonized evidence is scaled by a weight corresponding to each data stream. We group the supporting evidence versus the opposing evidence in layer 6, then we aggregate them in layer 7. The intersection between the supporting and opposing links flowing from layer 6 to layer 7 shows the amount of the decreased support reduced by the opposing evidence. Finally, layer 8 represents the produced $M_{\text{score}}$ and whether it is toward or against the new MoA–indication association.

As an example of the insights that can be derived from tracing the evidence transformation in this diagram, Figure 7 shows the diagram for the association between *anti-IL1RL2* and *Psoriasis*. The opposing portion of the transcriptomic evidence seems to balance the positive one, which leads to a nearly neutral contribution to the consensus score by this data stream. However, layer 2 shows that the actual amount of opposing evidence is small compared to the supporting one, indicating that the harmonization process skews the relative contributions of the positive and negative parts.

**FIGURE 8:**
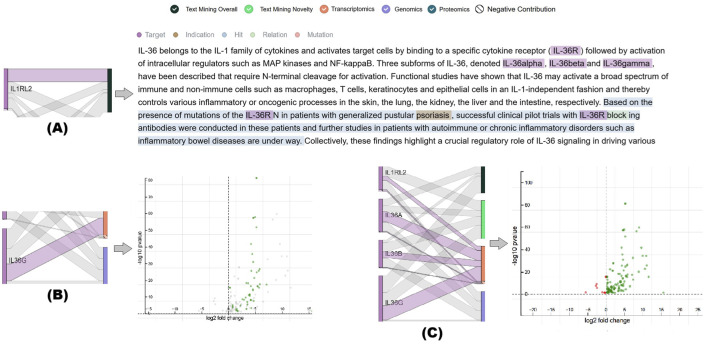
By interacting with the links between the target and data stream layers in the evidence flow diagram, users can drill down into the detailed qualitative evidence underlying the $M_{\text{score}}$ calculation. The figure shows several pieces of evidence for the association between *anti-IL1RL2* and *Psoriasis*. **(A)** An abstract retrieved by the text mining algorithm. Highlighted segments indicate the different aspects considered supporting the association by the algorithm and relevant to the *IL1RL2* receptor. **(B)** A volcano plot highlighting the parts of the transcriptomics evidence associated with the *IL36G* ligand. **(C)** Volcano plot with all the transcriptomics evidence highlighted due to selecting all links to the MoA targets.

For enhanced traceability of the evidence, the diagram displays the exact scalar value of the transformed evidence when hovering over the nodes in each layer. In addition, selecting a saturation function node in layer 3 plots the corresponding function for the specific data stream. To fully satisfy **DG3**, inline with **DG1** we enable evidence drill-down-analysis. Selecting the links between layer 1 and layer 2 enables users to drill down into a summary visualization of the collective qualitative evidence. For example, selecting the link between a molecular target and the transcriptomics stream node displays a volcano plot presenting the full transcriptomics evidence, with the pieces corresponding to the selected molecular target highlighted (see Figures 8b,c).

Furthermore, selecting a piece of the visualized collective evidence enables users to drill down to its corresponding qualitative representation. For example, Figure 8a shows the abstract of a selected publication analyzed by the text-mining algorithm. As illustrated in the same figure, at this level of analysis, we can also provide an instance-level explanation: the abstract is highlighted according to the specific aspects identified by the text-mining algorithm to support *anti-IL1RL2–Psoriasis* association. molIEreVIS highlights the abstract using annotation data generated by the text-mining algorithm itself.

#### 3.1.3 Validation stage

This stage functions as a consistency validation, allowing users to assess whether the MoA-disease association is compatible with broader existing curated knowledge, thereby addressing **DG4**. To this end, we employ PrimeKG (Chandak et al., 2023), a comprehensive knowledge graph that, at the time of publication, integrates 20 high-quality resources to describe 17,080 diseases with 4,050,249 relationships. It represents ten major biological scales including disease-associated protein perturbations, biological processes and pathways, anatomical and phenotypic scale, and the entire range of approved and experimental drugs with their therapeutic action. PrimeKG inevitably reflects existing literature biases. However, because this stage operates within a human-in-the-loop workflow rather than as an automated decision rule, such biases can be consciously accounted for by experts. For example, underrepresentation of first-in-class MoA would not lead to outright rejection of a candidate, but could instead be interpreted as a potential gap in current knowledge.

Inspired by previous work (Wang et al., 2022; Nunes and Pesquita, 2024; Jiang et al., 2025), we chose a *path-based representation* for the MoA–indication connectivity: we display independent sequences of entities connecting the MoA to the indication, rather than showing a full *node-link diagram*. These sequences are subgraphs that connect an MoA linearly with an indication via intermediate nodes. We chose this design because the path-based representation aligns more closely with experts’ mental models and the way they communicate their findings, which was also noted by Wang et al. (2022) who empirically demonstrated its efficiency compared to the node-link diagram.

We designed this stage as a query interface for PrimeKG. The user can build two-sided queries: the left side represents a selected MoA, and the right side represents an indication to validate. The query retrieves the shortest paths between the two sides. Since PrimeKG does not include MoA nodes directly, we use the molecular targets of the MoA as the left side of the query. The user can optionally exclude any of these targets from their query.

We represent query results as *meta-paths*. A meta-path is a sequence of node and edge types that groups semantically similar paths. In line with **DG1**, our meta-paths are expandable. When expanded, a meta-path reveals the paths with actual entities and links in PrimeKG that follow its structural pattern. Because the number of such paths can be large, we paginate them and allow users to optionally define the page size. In line with Wang et al. (2022), we visually distinguish between a *type node* (nodes that represent a type of entity, such as protein or anatomy) in a meta-path and an actual *entity node*, by representing type nodes as outlined rectangles and entity nodes as filled rectangles. Each of the 20 node types in PrimeKG is assigned a unique color. Type nodes in meta-paths provide filters that enable users to sharpen their exploration, focusing on a subset of the grouped paths. Meta-paths are displayed in descending order based on the number of paths they group, and users have the option to change this order.

**FIGURE 9:**
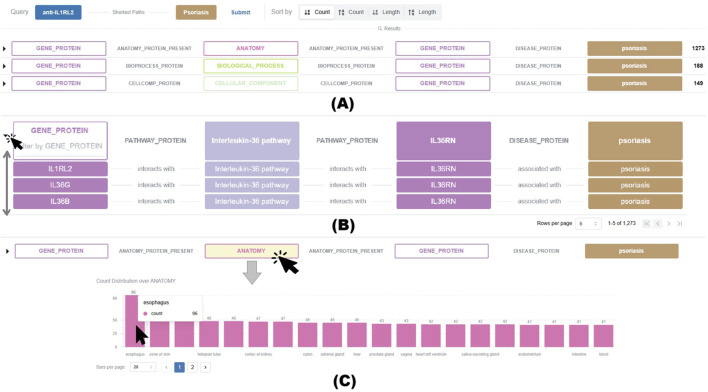
**(A)** A sample query and several of the retrieved meta-paths. The number of actual paths grouped under each meta-path is shown on its right end. **(B)** An example of an expanded meta-path, showing the paginated grouped paths and the filter input available for each type node. **(C)** A paginated distribution chart summarizing the contribution of different anatomies to the paths grouped under a single meta-path. Hovering over the bar displays the entity name and its count.

Figure 9a illustrates a query for paths connecting *anti-IL1RL2* with *Psoriasis*, and several of the retrieved meta-paths. Figure 9b illustrates an expanded meta-path showing how *IL1RL2*, *IL36G*, and *IL36B* interact with the *Interleukin-36* pathway, a pro-inflammatory signaling cascade implicated in psoriasis. This meta-path supports the role of *IL1RL2* as the receptor mediating *IL-36*–driven inflammatory responses in psoriatic pathology. In addition, as shown in Figure 9c, when a type node in a meta-path is selected, molIEreVIS displays a distribution chart that summarizes the PrimeKG entities grouped under this type node. For example, the distribution chart in Figure 9c reveals that esophagus is the most prevalent anatomy entity in this meta-path.

### 3.2 User study results

In this section, first, we highlight the usability difficulties we observed in the current design. Second, we summarize our observations during the open-ended exploration. Finally, we present the feedback collected from the post-study open-ended questions.

#### 3.2.1 Usability difficulties

Most of the hesitation was observed in the exploration stage, in particular during the atomic tasks when participants engaged with the weight comparison interface. Participants either forgot a required step for completing the interaction, triggering an intervention on our side, or became confused for a moment when adding a new weight set, adjusting it, or making it the reference set. In contrast, during the open-ended task, participants overcame their hesitation and interacted more smoothly with the interface. This pattern suggests that while the current design is ultimately learnable, it is not immediately intuitive and would benefit from improved procedural clarity. In addition, all participants overlooked indications sharing the same rank in the ranking table, suggesting a need for a visual encoding to make this situation more perceptible. Some difficulty was also observed in the engagement with the MeSH ontology. In particular, **P3**, who is least familiar with the molIEre pipeline processes, hesitated when asked how to incorporate the $M_{\text{score}}$ distribution sparklines in their analysis. Participants **P1** and **P2** interacted more smoothly with this interface, and **P2** stated *“The distribution histogram is helpful to quickly get an idea of which disease groups to check out further”*. The only usability issue we observed during the validation stage was that participants found it tedious to deselect the irrelevant disease names suggested by the fuzzy matching. No noteworthy usability difficulties were observed during the interpretation stage.

#### 3.2.2 Open-ended exploration

Overall, all three participants demonstrated a clear understanding of the workflow after completing the onboarding and atomic tasks. They were able to interact smoothly and meaningfully with molIEreVIS, interpret its visual cues, obtain insights, and integrate information across the different stages to assess plausible indications. During the open-ended exploration, the exploration stage proved to be a good entry point for the analysis. Participants found it to be integrated well with the following stages, and to align with their typical assessment workflow. One participant found the original indication for an MoA among its top-ranked indications, stating *“That’s a good sign! I’m happy with the rankings”* (**P2**) . **P3** looked for one indication they had in mind in the ranking table, which turned out to have a low rank and lack evidence. Upon checking the validation stage, the participant found the paths relatively long and characterized by negative relationships, and commented *“I’m not surprised”* (**P3**).

Interestingly, participants spent most of their open-ended exploration time in the interpretation stage. All participants drilled down to the underlying evidence and appreciated the integrated access to the diverse data sources, especially the convenient access to publications and their abstracts collected through the text mining stream. All participants also liked the highlighted segments in the abstracts that indicate the different aspects considered by the text mining algorithm. **P1** appreciated the visual clarity of the evidence flow diagram in distinguishing supporting from opposing evidence, a distinction that she described as *“harder to see in our deep dives”*. **P1** wanted to compare the evidence flow diagram and its underlying evidence for two indications. As our current design does not support direct comparison, she moved back and forth between evidence charts for two indications, observing that *“This indication is supported by more publications, but the other seems to be supported by more novel ones”* (**P1**). She expressed interest in facilitating the comparison: *“I’d have liked more comparison features”* (**P1**).

In the validation stage, **P1** was surprised to find only one path connecting the selected MoA and the candidate indication, but stated: *“Interesting to look at this path, I would definitely have missed this”* (**P1**) . **P2** explained her reasoning for examining the paths: *“The length of the paths and intermediate nodes can tell you how well something is studied”* (**P2**). Across participants, we observed a strong reliance on the distribution charts to identify dominant biomedical entities under a node type, which they preferred over using the filtering feature. While **P1** and **P2** expressed enthusiasm about further exploring the validation stage, both **P1** and **P3** felt that this stage was overloaded with information. Additionally, **P3** expressed interest in being able to construct more flexible queries within this stage.

#### 3.2.3 Feedback

All participants agreed that molIEreVIS would be a valuable addition to their typical workflow, by enabling rapid identification of promising indications for further investigation: *“Quick checks can be easily performed for indications”* (**P1**). They also reported feeling well supported in their analyses, particularly when leveraging the interpretation stage. **P2** suggested two additional extensions that would make her feel even more supported: the ability to combine and export multiple visualizations into a compact “disease card” summarizing findings for a given indication, and the incorporation of LLMs to generate summaries of the evidence.

## 4 Discussion

In this section we reflect on learnings from the user study and derive interesting avenues for future work from our findings.

### 4.1 Ranking list and MeSH interface

Overall, molIEreVIS was well received by our small group participants. However, as described in Section 3.2.1, we also noticed some parts of the workflow that could have been smoother. In particular, we were surprised that the exploration stage seemed to cause the most confusion. Being based on a simple ranking table and a selection interface using the MeSH disease ontology, we deemed this stage the most straightforward in terms of usability. During the user study, we identified two potential improvements.

One, users got confused by the discrepancy between the ranking table and the MeSH tree. The ranking table lists only terms for which the pipeline had been run, and the terms are presented in a flat list. Here, items appear flush left regardless of the level at which they would appear in the MeSH hierarchy. In contrast, in the MeSH tree view, all MeSH terms are present, but the ones for which no pipeline data is available cannot be selected. A future version of molIEreVIS could attempt to incorporate the MeSH hierarchy directly in the ranking. We considered such an approach during the design phase, but ultimately decided to split the interaction between the simpler, flat table and the dedicated MeSH view.

Two, the modification of weights and the comparison of rankings caused some confusion. While we attribute this in part to some of the participants not being familiar with molIEre processes, we are still trying to understand how the UI could be improved to better support this task. Ranking comparison involves the creation of a new set of weights, the definition of a reference set of weights, and understanding the visualized ranking differences. To improve this workflow, we originally considered the introduction of bump charts to more explicitly encode ranking changes, but found them to clash with the compact horizontal stacking of the $M_{\text{score}}$ charts. We chose this stacking in the first place due to its suitability for comparison. It remains a challenge to find a more intuitive workflow for the ranking comparison task in molIEreVIS—even though it must be noted that we expect a limited number of users to often experiment with different weights.

### 4.2 Evidence flow chart

We were delighted by how intuitively all users interacted with it, even though it included several custom encodings that users could not have been familiar with from other tools. In particular, the changing node scale throughout the pipeline steps and the encoding of negative values, which typically cannot be represented in standard Sankey charts, did not confuse any of the study participants.

Still, one of the takeaways from the user study concerns the evidence flow chart and its use in the interpretation stage. As stated in Section 3.2.2, one participant mentioned that they would have liked better comparison features during this stage. With the current design, a comparison of two diseases for the same MoA could be achieved by placing the molecular target nodes at the center and having two copies of the chart—one for each disease—fanning out to either side. To further facilitate comparison, it might be necessary to allow users to collapse parts of the chart to bring nodes and edges to be compared closer together. A dedicated comparison would also require a vertical alignment of nodes across diseases. It is not obvious how the current design can be directly used for comparing more than two diseases, other than simply juxtaposing multiple linked evidence charts.

### 4.3 Path exploration

Our participants felt overwhelmed by the amount of information shown during the validation stage. We attribute this observation to the fact that the participant’s current workflow is not based on paths through a dense knowledge graph like PrimeKG. Moreover, in some cases, many hundreds or thousands of “shortest” paths can exist, with dozens of different meta-paths. In those cases, it might be beneficial not to show all available information but to introduce further abstraction layers. These abstraction layers can take the form of visual summaries, each conveying a specific aspect of all paths instantiated by a given meta-path. Such representations enable users to reason about the global structure before drilling down into individual paths. For example, an under-planning representation visualizes a meta-path as a conditional relationship between entity sets defined by the query endpoints, for instance the relationship between the set of disease-associated genes and the set of anatomical contexts in which a gene is expressed.

The recent work by Jiang et al. (2025) demonstrates how LLMs, when combined with knowledge graphs, can assist experts in iteratively exploring, reasoning about, and refining hypotheses derived from complex knowledge graph paths. Incorporating LLMs could also be a promising direction in molIEreVIS to mitigate information overload. In a related research project on knowledge graph curation, we gained positive experience with a customized LLM chat that has access to the selections and encodings of a visual interactive tool. However, it is crucial to keep the interaction between the LLM and the user collaborative rather than fully autonomous to maintain reliability and prevent hallucination.

In addition, inspired by Partl et al. (2016), we are already developing an improved visual query editor that allows users to flexibly filter the paths shown, which should improve the process of finding individual paths or groups of paths of interest, even when there are many meta-paths.

### 4.4 Different data contexts and additional data streams

The visualization approach resulting from this study is grounded in a conceptual model of multimodal drug repurposing pipelines (see Table 3). This abstraction enhances adaptability in different data, and pipeline contexts beyond the specific instantiation demonstrated in this paper. In this section, we discuss this adaptability in more detail.

As for the implementation scope specific to the context of molIEre, the demonstrated components of molIEreVIS are modular and can be substituted as needed. For instance, although we use PrimeKG in the validation stage, any knowledge graph can be used instead. The same flexibility applies to the choice of disease ontology that, in our demonstration, is MeSH. Moreover, additional data streams can, in principle, be integrated into the workflow. In practice, a new data stream requires the development of corresponding collective and individual piece of evidence charts, that will be shown when a link corresponding to evidence of the newly added data modality is selected in the evidence flow chart. In terms of visual scalability, we expect the evidence flow chart to easily scale to about twice as many data streams as are implemented in the prototype version.

As discussed in Section 2.2, beyond molIEre different pipelines may realize evidence transformation boundaries in different ways. In particular, some multimodal pipelines collapse multiple boundaries by embedding heterogeneous evidence into a shared latent representation and performing evidence integration within a single black-box model. In such cases, the intermediate data artifacts associated with the collapsed boundaries are no longer explicitly available. Accordingly, **DG3** reflects an intentional abstraction that avoids exposing these collapsed data artifacts, namely low-level model states, which would not align with the target users’ understanding of the pipeline or their reasoning model.

The integration boundary is essential for the multimodal drug repurposing pipelines. However, some pipelines may not explicitly expose per-stream contributions. In such cases, **DG3** recommends estimating these contributions, as they constitute an important data artifact in expert reasoning model. One way to restore the interpretability can be by computing feature attributions with established methods and grouping them by evidence stream.

Additionally, the instance-level explanations used for the literature-based evidence stream in molIEreVIS can be understood more generally as a drill-down mechanism for evidence investigation. In pipelines where evidence is transformed using model-based approaches, a comparable visualization could be supported through established attribution methods that relate model outputs back to individual evidence instances.

“What-if” analysis **DG5** remains feasible in collapsed-boundary settings by operating at the level of evidence rather than model internals. Such analysis can be enabled by perturbing semantically meaningful groups of input evidence (e.g., entire data streams or subsets), for example by selectively removing, substituting, or reweighting them. This allows users to explore alternative scenarios and assess the robustness of results without requiring direct control over the collapsed transformation boundaries.

Finally, although this work primarily addresses decision support for drug repurposing, as noted in Section 2.2, the conceptual model of multimodal pipelines on which our study is grounded has also been observed in other drug discovery domains, such as target prioritization and biomarker discovery. These domains serve as potential directions for extending the adaptability of this study.

### 4.5 Additional features

In our open discussions toward the end of the user study sessions, participants mentioned two concrete ideas for future extensions of molIEreVIS.

One, experts sometimes prepare so-called disease cards to present and document the findings of the assessment process in a compact form. It should be relatively straightforward to include a mechanism for exporting selected charts or publication details for use in such disease cards. However, it is not clear how to merge the findings from molIEreVIS efficiently with evidence users might have found elsewhere. This might be necessary because users mentioned that they found it likely to incorporate molIEreVIS in their workflows, but that they do not want to completely abandon their existing practices in its favor.

Two, users commented that they would appreciate automatic textual summaries of evidence directly within molIEreVIS. In the future, we would like to experiment with LLM-based summaries (Xu et al., 2024; Choi et al., 2025) of the various charts, in particular, the summary plots accessible through the evidence flow chart. The text-mining evidence may also serve as a rich source for textual summarization.

While we are eager to explore this further, we realize the risk of hallucinations in the context of evidence analysis. Therefore, the expert should remain the final decision maker, using the LLM output as contextual guidance rather than authoritative conclusions.

## 5 Conclusion

In this paper, we presented an interactive visualization approach designed to support experts in evaluating drug repurposing opportunities prioritized by a computational pipeline that integrates evidence from multimodal data sources. Grounded in a design study, our approach abstracts domain experts’ practices into a staged reasoning workflow comprising exploration, interpretation, and validation. We also demonstrated molIEreVIS, which instantiates our approach within an operational multimodal drug repurposing pipeline. molIEreVIS exposes candidate rankings, evidence provenance across transformation boundaries, and knowledge graph context in a coordinated manner. Experts’ feedback on molIEreVIS highlights the potential for improving computational drug repurposing workflows through interactive visualization.

## Data Availability

Public datasets have been used in the prototype implementation. These datasets can be found here: https://github.com/mims-harvard/PrimeKG and here: https://www.nlm.nih.gov/databases/download/mesh.html.

## Footnotes

1
https://www.boehringer-ingelheim.com
