# Supplementary material for: molIEreVIS: exploring and interpreting the evidence behind drug repurposing predictions
Source: Front Bioinform. 2026 Mar 30;6:1756459. doi: 10.3389/fbinf.2026.1756459 (PMC13071390; doi:10.3389/fbinf.2026.1756459)
Supplement: Supplementary file 1 [file DataSheet1.pdf]

User Study Feedback Questions

Researchers used the following questions to steer the semi-structured discussion with the participants before the closure of the study sessions:

q1. If you had to explain your decision to your team using this tool, would you feel well-supported?

q2. Would the tool help you confidently prioritize an indication?

q3. How would you compare this experience to your regular workflow?

q4. Did the tool help you notice anything you might have missed without it?

q5. Would you want to incorporate this tool in your regular workflow?

q6. What would you change to make this tool more satisfying?
